# Quantitative measurement of density fluctuations with a full-field laser interferometric vibrometer

**DOI:** 10.1007/s00348-019-2842-y

**Published:** 2019-11-28

**Authors:** Felix Greiffenhagen, Jakob Woisetschläger, Johannes Gürtler, Jürgen Czarske

**Affiliations:** 1 Institute for Thermal Turbomachinery and Machine Dynamics, Graz University of Technology, Inffeldgasse 25A, 8010 Graz, Austria; 2 Laboratory of Measurement and Sensor System Technique, Faculty of Electrical and Computer Engineering, Technische Universität Dresden, Helmholtzstr. 18, 01062 Dresden, Germany

## Abstract

**Abstract:**

Modern, lean and premixed gas turbine combustion concepts for low NO_x_ emissions are prone to combustion instabilities. In a previous work it was shown that laser interferometric vibrometry (LIV) can be used to record global as well as local heat release fluctuations in swirl-stabilized premixed methane flames quantitatively, if other effects influencing density are small. In this work a newly developed camera-based full-field LIV system (CLIV) was applied to a lean, confined, premixed and swirl-stabilized methane flame under atmospheric conditions. Instead of time-consuming pointwise scanning of the flame, CLIV records full-field line-of-sight density fluctuations with high spatio-temporal resolution. With a recording rate of 200 kHz, CLIV enables the visualization of highly unsteady processes in fluid dynamics and combustion research. As an example for an unsteady process, the propagation of the flame front through a lean, premixed gas volume is visualized during an ignition process. A discussion of algorithms and assumptions necessary to calculate heat release oscillations from density oscillations is presented and applied to phase-averaged data recorded with CLIV for this type of flame. As reference, OH* chemiluminescence data were recorded simultaneously. While density gradients travelling with the flow are recorded by LIV and CLIV, chemiluminescence imaging will show nothing in the absence of chemical reaction.

**Graphic abstract:**

**a** Time-averaged density gradient within the combustor in lateral direction. **b** Density fluctuations along line-of-sight 7 ms after ignition. **c** Phase-averaged and local heat release fluctuations at 225 Hz perturbation frequency
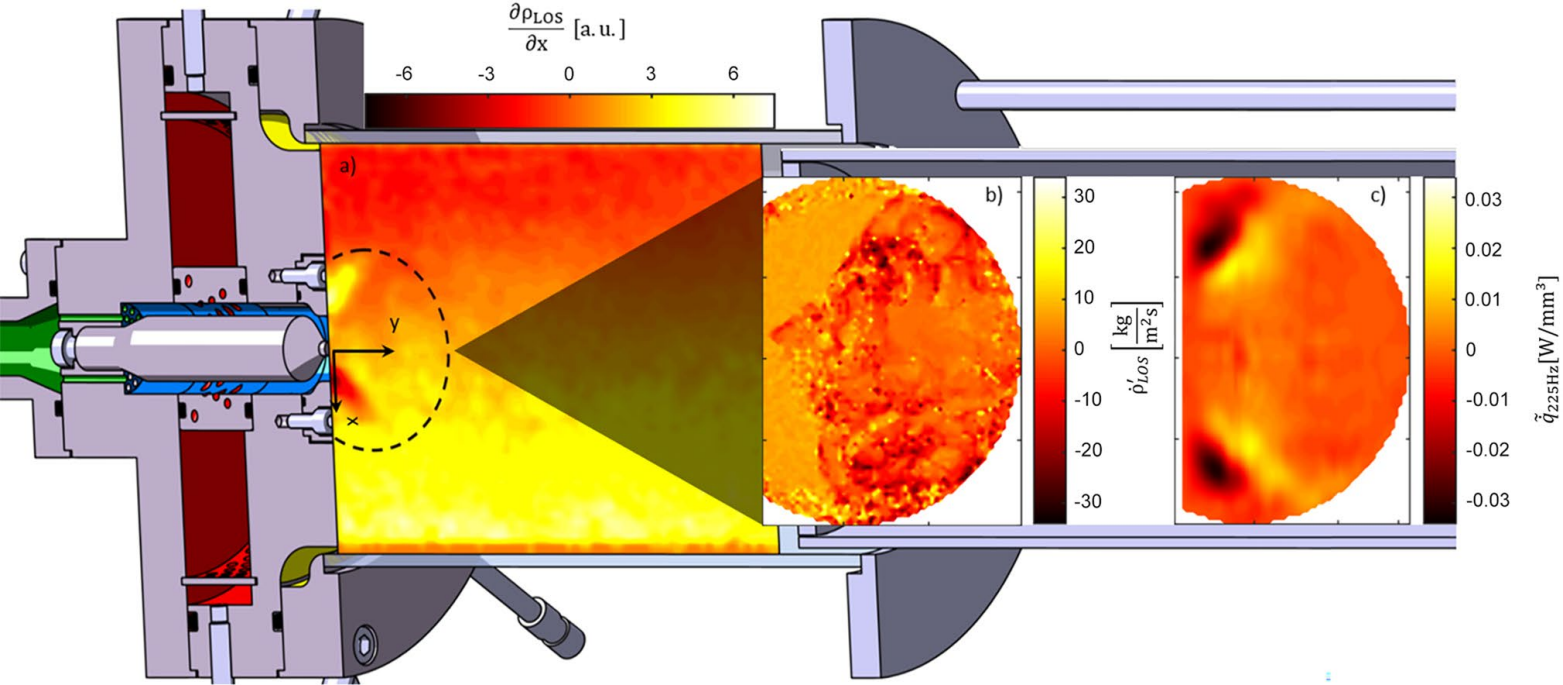

## Introduction

Due to reduced core mass flow and damping capabilities, modern low NO_x_ combustion concepts, operating at lean and premixed conditions, are more vulnerable to instabilities (Dowling and Yasser 2015). Pressure fluctuations caused by unsteady heat release are not only a source for combustion noise, but in a worst case can lead to malfunction or even damage of gas turbines. Contrary to this, improvements of the acoustics of fan, compressor and turbine reduced the individual contribution of these components putting the combustor more into focus. In the frequency range of approximately 200–2000 Hz, the combustion process is the main contributor on the overall engine noise (Dowling and Yasser 2015).

Swirl-stabilized flames used in modern combustion concepts, tend to react nonlinear to incoming perturbations (Candel et al. 2014) from the fuel or air path. The complex interaction between highly turbulent and unsteady swirled flow fields, chemical reaction kinetics and acoustics in a field of strong temperature gradients still requires experimental investigations to quantify the stability behavior of a combustion system. If optically accessible, common experimental approaches are OH* or CH* radical chemiluminescence or planar laser-induced fluorescence (P-LIF). According to Balachandran et al. (2005), chemiluminescence can be used as measurement for global heat release in premixed flames with a constant equivalence ratio. However, since the emission of radicals is influenced by strain rate and mixture gradients, Lauer (2011) recommends to carefully interpret local chemiluminescence data.

A more recent experimental approach is the recording of density fluctuations with laser interferometric vibrometry (LIV) in unconfined (Giuliani et al. 2006; Leitgeb et al. 2013; Fischer et al. 2013) as well as in confined flames (Peterleithner et al. 2016). Such commercially available LIV systems were originally designed to record surface vibrations but can also be used to detect integral changes of the refractive index along the laser beam. The line-of-sight time derivative of the refractive index then can be correlated to density fluctuations. There are several sources which may change density, such as fluctuations in heat release or pressure and turbulence, but also density gradients travelling with the flow will be detected as fluctuations in density via LIV. If other sources are neglectable density fluctuations can be used to calculate local and global fluctuations in heat release (Greiffenhagen et al. 2019). Via tomographic reconstruction also local data can be derived with high accuracy. Due to the high sensitivity of modern LIV systems, changes of the optical path length in the range of nm/s can be resolved, which allows to detect acoustic waves around machines or instruments (Zipser et al. 2002; Gren et al. 2006) or density fluctuations in turbulent flows (Mayrhofer and Woisetschläger 2001; Martarelli et al. 2013).

Considering this, LIV seems an interesting alternative or extension to chemiluminescence when the stability behavior of swirl-stabilized flames is analyzed. It offers global as well as local data and even pressure waves surrounding the flame can be visualized. However, time-consuming scanning of the measurement volume is a big disadvantage compared to chemiluminescence, since only single positions in the flame can be recorded within one measurement. Depending on the requirements in terms of spatial resolution, the beam diameter is from 2 to 5 mm, resulting in up to 1000 measurement positions, with a sample time of 60 s each to record the whole flame. This not only leads to long measurement time for a single projection, in which the operation conditions of the flame must not change but also prohibits full-field measurement of unsteady processes.

To overcome those limitations, a planar, camera-based full-field-LIV system (CLIV) was developed. The system presented here is capable of measuring the whole combustion zone in a field of approximately 51 × 51 mm^2^ (110 × 110 px), with a lateral resolution of 0.47 × 0.47 mm^2^ each and at a frame rate of 200 kHz instantaneously. The refractive index fluctuations obtained from CLIV are used to calculate heat release fluctuations in a swirl-stabilized, confined, premixed and lean 3.44 kW model combustor, operated at atmospheric conditions. To record the transfer function of the combustor, a siren was used to excite the flame at defined frequencies. A quantitative comparison of fluctuation amplitudes and phase angles, obtained from OH* chemiluminescence and recorded simultaneously with a photomultiplier (PM), was carried out. Finally, time-averaged local data and time-resolved data of an unsteady ignition process are shown.

## Background

### Linking heat release and density

Interferometry is widely used to detect refractive index and variations thereof. Using the Gladstone–Dale relation, refractive index oscillations can be linked to oscillations in density. In the next paragraphs a short overview is given, how fluctuations in density, heat release and pressure are linked to each other. A more detailed discussion can be found in Greiffenhagen et al. (2019).

Changes in density can be caused by superposition of numerous phenomena. The full relation between these numbers in a reactive flow, can be derived from the energy equation as shown by Williams (1994, Crighton et al. 1996 and Lieuwen 2012). A first simplification of the basic equations is possible, when hydrocarbons are burned in air under lean conditions, which allows to assume constant molecular masses (Lieuwen 2012). In a further step the heat losses by radiation and conduction to the walls, as well as heat addition due to viscous effects can be neglected. Measurements with a thermopile showed, that heat losses by radiation (wavelength between 0.2 and 50 µm) were in the range of five percent of the total thermal power. Viscous effects are assumed to be neglectable due to low Mach numbers. Also conduction to the walls is low for stable points of operation. Considering this, density fluctuations can be written as function of fluctuations in pressure and heat release:

1 $$\frac{{{\text{d}}\rho }}{{{\text{d}}t}}\; = \; \frac{\rho }{p}\frac{{{\text{d}}p}}{{{\text{d}}t}}\; - \; \frac{{\left( {\gamma - 1} \right)\rho }}{\gamma p}\frac{{{\text{d}}q_{v} }}{{{\text{d}}t}}$$

with $\rho$ representing density, $p$ pressure, $\gamma$ the ratio of heat capacities and $\frac{{{\text{d}}q_{\text{v}} }}{{{\text{d}}t}}$ the volumetric heat release. Considering that fluctuations $\rho^{\prime}$ of the flow around a steady state $\bar{\rho }$ are only small relative to the steady state, for pressure, density and the ratio of heat capacities it can be assumed that $p \approx \bar{p}$, $\rho \approx \bar{\rho }$ and $\gamma \approx \bar{\gamma }$. The substantial derivative of density $\frac{{{\text{d}}\rho }}{{{\text{d}}t}}\; = \;\frac{\partial \rho }{\partial t} + \left( {v\nabla } \right)\rho$ consists of a local time dependent term and a convective part $\left( {\varvec{v}\nabla } \right)\rho$. Using a single-point LIV system (Greiffenhagen et al. 2019), thermoacoustic oscillations had to be excited by a siren to enable spatio-temporal correlations between the scanned positions. Such a triggered scanning LIV is necessary to obtain the convection term in above equation. These terms also provide information on the dynamics of the system. It is important to note that a high-speed camera-based LIV system (CLIV) enables spatio-temporal correlations without siren trigger since the single pixel signals are recorded simultaneously.

When studying thermoacoustic low Mach number problems, it is common practice, to replace the substantial derivative in Eq. (1) by partial derivatives. Thus, it is assumed that the driving potential of thermoacoustic instabilities is purely from the local heat release fluctuation (Peterleithner et al. 2016). Under these assumptions, Eq. (1) reads:

2 $$\dot{q}_{v}^{\prime } = - \frac{{\bar{\gamma }\bar{p}}}{{\left( {\bar{\gamma } - 1} \right)\bar{\rho }}}\frac{{\partial \rho^{\prime } }}{\partial t}$$

with $\dot{q}_{v}^{\prime }$ [W/m^3^] denoting the heat release fluctuations per unit volume. As can be seen from Eq. (2) the factors $\bar{\rho }$ and $\bar{\gamma }/(\bar{\gamma } - 1)$ are scaling the results, therefore local information on these numbers is important to calculate accurate data. Thus, local data for $\bar{\gamma }/(\bar{\gamma } - 1)$ enhance the accuracy in a range of seven percent. If the influence of mean density in the combustion zone is compared to local data when calculating the heat release oscillations, results differ in the range of 5–10 percent. Two measurement methods provided the necessary data, namely background-oriented schlieren (BOS) technique records mean local density or temperature, and spectroscopy records local equivalence ratio. The latter is also used to calculate the species-dependent local Gladstone–Dale constants, relating density and refractive index (Greiffenhagen et al. 2019).

Equation (1) shows, that the relations between density, pressure and heat release fluctuations are linear. In 300 mm distance from the unconfined flame 70 dB were recorded at the siren frequency. Assuming a sound pressure level of 100 dB in the confined flame, the pressure-caused density fluctuations are three orders of magnitude smaller than the total density fluctuations recorded by LIV in the flame. Thus, pressure fluctuations must be in the 1 kPa range to reach the same magnitude as heat release fluctuations in the reaction zone. For pressure fluctuations such high Eq. (1) must be considered, the assumptions for Eq. (2) are no longer valid. The same applies to measurement positions outside the reaction zone, where not heat release occurs. But since oscillations in density measured in those regions are low, the impact on global values can be estimated as low.

According to combustion noise theory, the second time derivative of density fluctuations integrated over the flame volume $V_{\text{F}}$ results in a signal which is proportional to the acoustic pressure fluctuation in the far field $p_{\text{farfield}}^{\prime }$ of an unconfined flame. This was first proposed by Strahle 1971 and tested by the authors using a commercially available single-beam LIV(Greiffenhagen et al. 2017):

3 $$- \int\limits_{{V_{\text{F}} }} {\frac{{\partial^{2} \rho^{\prime } }}{{\partial t^{2} }} {\text{d}}V \propto p_{\text{farfield}}^{\prime }}$$

Thus local density fluctuations are essential for the investigation of thermoacoustic oscillations. In combination with other techniques (e.g., chemiluminescence) the influence of direct and indirect noise can be discussed (Greiffenhagen et al. 2019; Leitgeb et al. 2013).

### Design of a camera-based laser interferometric vibrometer

**Fig. 1:**
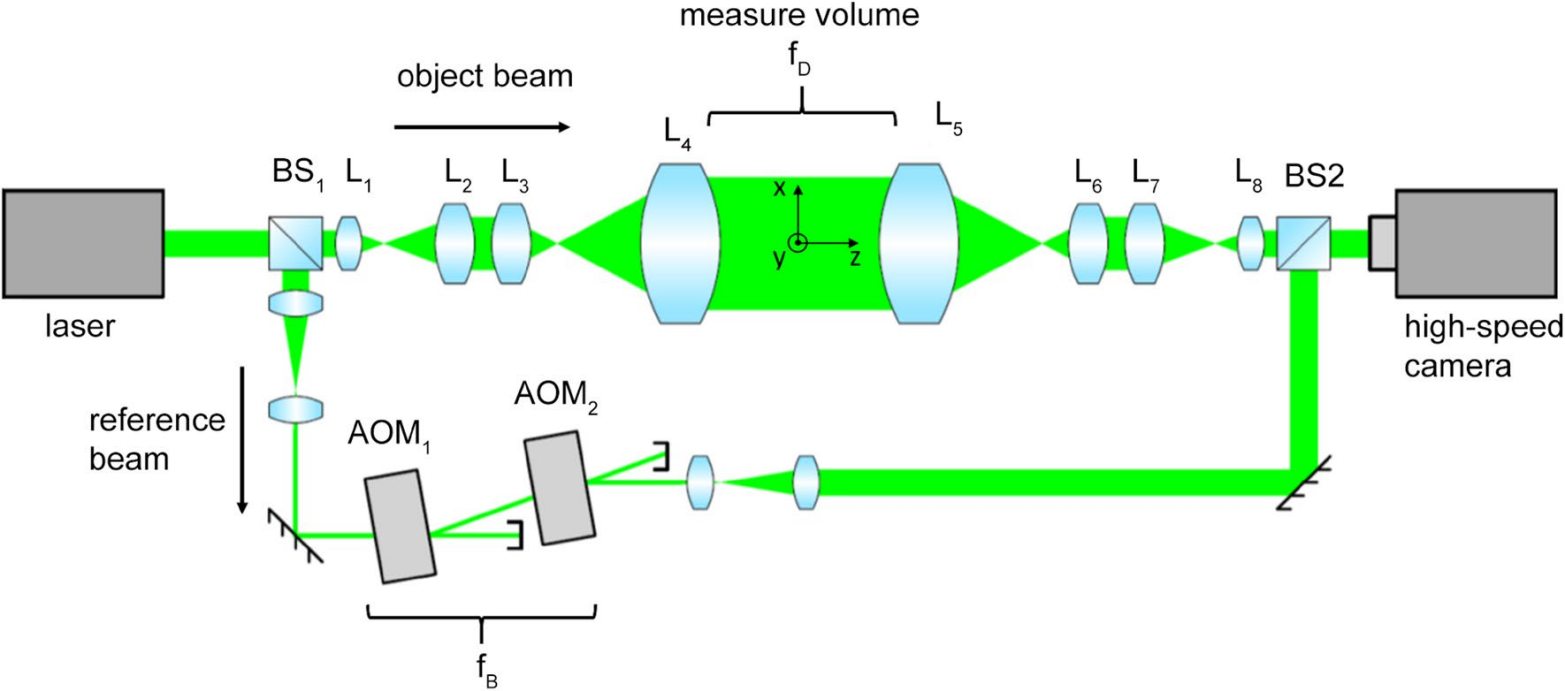
Top view on CLIV setup: the laser beam is split up into a reference and an object beam by beam splitter 1 (BS1). Lenses L1–L4 enlarge the object beam to a diameter of 71 mm. Behind the measurement volume lenses L5–L8 reduce the object beam diameter again. The reference beam is guided around the flame at a safe distance and modulated with two acousto-optical modulator AOM1 and AOM2 to create a carrier frequency $f_{\text{B}}$ of 50 kHz. After beam splitter 2 (BS2) both beams are interfering at the image sensor of a high-speed camera

In general, an interaction of light with any fluid leads to delayed wave fronts, based on a reduction of the speed of light, characterized by the refractive index $n$. The refractive index depends on the species present in the fluid and its density, where the link between density and refractive index can be expressed via the Gladstone–Dale relation (Gardiner et al. 1981; Merzkirch 1987). By superimposing a reference beam with an object beam, as depicted in Fig. 1, interferometry is a common way to measure the refractive index, based on the difference of the optical path length $L = \smallint n\;{\text{d}}z$ between the two beams. If the geometrical path is kept constant, such systems can be used to detect changes of the optical path length with high precision in the range of nm/s.

Since commercially available LIVs use a single detector to record the interference pattern (Lewin 1998), time-consuming scanning of the volume of interest is necessary to achieve a desired spatial resolution. Contrary to this, CLIV uses a high-speed camera (Phantom v1610, Vision Research) with a resolution of 110 × 110 px, a pixel size of 28 µm and a frame rate of 200 kHz to record the interference pattern. Similar to an array of 12,100 individual, simultaneous LIVs, CLIV records the whole measurement volume synchronous.

The camera-based full-field LIV presented in Fig. 1 is based on a Mach–Zehnder type interferometer (Gürtler et al. 2017). The intensity

4 $$I\left( t \right)\; \cong \;1\; + \;V \cos \left( {\Delta \phi \left( t \right)} \right)\quad {\text{with}}\;V \in \left[ {0,\;1} \right]$$

detected on the camera sensor depends on the modulation depth $V$, based on the intensity ratio and the angle between the laser beams and oscillates due to the time dependent phase difference $\Delta \phi_{\text{homodyne}} \left( t \right) = \frac{2\pi L\left( t \right)}{\lambda }$, with $\lambda$ the laser wavelength. To not only detect the absolute value of $L$, a heterodyne setup is used and, thus, the frequency of the reference beam is shifted by the carrier frequency $f_{\text{B}}$ using two acousto-optical modulators (Bragg-cells). The intensity signal on the camera now oscillates with the time-dependent frequency $f_{\text{I}} \left( t \right) = \frac{{{\text{d}}\Delta \phi_{heterodyne} \left( t \right)}}{{2\pi {\text{d}}t}}\; = \;f_{\text{B}} \; + \;\frac{{{\text{d}}L\left( t \right)}}{{\lambda {\text{d}}t}}\; = \;f_{\text{B}} \; + \;f_{\text{D}} \left( t \right)$, with the Doppler shift $f_{\text{D}}$. Using the definition of the optical path length and the Gladstone–Dale relation, the Doppler shift can be expressed as a function of the time derivative of density

5 $$f_{\text{D}} = \frac{1}{\lambda }\int {\frac{\text{d}}{{{\text{d}}t}}n\left( {x,y,z,t} \right){\text{d}}z} = \frac{G}{\lambda }\int {\frac{\text{d}}{{{\text{d}}t}}\rho \left( {x,y,z,t} \right){\text{d}}z = \frac{G}{\lambda }\frac{\text{d}}{{{\text{d}}t}}\rho \left( {x,y,t} \right)_{\text{LOS}} }$$

where $x$ and $y$ are the pixelwise coordinate of the camera frame, $G$ is the Gladstone–Dale constant and the index ${\text{LOS}}$ denotes a line-of-sight integration along the laser beam axis (z-axis). Hence, the evaluation of the instantaneous frequency of the camera signal is necessary to determine the change of density inside the measurement volume. This evaluation is done by removing the constant component of the intensity signal and applying quadrature demodulation technique to achieve the instantaneous phase $\Delta \phi \left( t \right) = \arctan \left( {\frac{{{\mathcal{H}}\left\{ {I\left( t \right) - \bar{I}} \right\}}}{{I\left( t \right) - \bar{I}}}} \right)$, where ${\mathcal{H}}$ denotes the Hilbert transform, with ${\mathcal{H}}\left\{ {V \cos \left( {\Delta \phi \left( t \right)} \right)} \right\}\; = \;V\;\sin \left( {\Delta \phi \left( t \right)} \right)$.

The principal scheme of the actual measurement system is shown in Fig. 1. On the left side, a 532 nm narrowband laser (Cobolt Samba 532) is separated into an object and a reference beam with a beam splitter (BS1). The object beam is expanded using the lens system L_1_–L_4_, resulting in a collimated beam with a diameter of 71 mm. After passing the measurement volume, the beam is imaged onto the camera sensor, using the telecentric lens setup L_5_–L_8_. This is necessary to avoiding cross talk between pixels, caused by refractive index gradients or fluctuations thereof along x- and y-directions. When a telecentric system is used, any beam distortion due to refractive index gradients inside the measurement volume results only in an angle variation of the incident beam on the sensor and therefore no cross talk. To avoid the superposition of light emission of the flame with the laser, a band pass filter for a wavelength of 532 nm was used in front of the camera. The reference beam is guided around the measurement volume at safe distance, so that no refractive index changes, which may alter the reference beam, can occur. The carrier frequency $f_{\text{B}}$ is realized by a cascade of two acousto-optical modulators (AOM), wherein the dependence of the frequency shift on the direction of incidence of the laser beam is utilized. AOM_1_ is operated at the center frequency $f_{\text{C}}$ = 200 MHz and AOM_2_ with the frequency $f_{\text{C}} + f_{\text{B}}$, so that after passing both AOMs the carrier frequency $f_{\text{B}}$ modulates the reference beam. When setting the carrier frequency, following considerations have be taken into account:

1. The expected frequency range of the oscillating density inside the flame is about 1 kHz, yet higher harmonics of each component occur in the intensity signal due to the nonlinearity of the cosine function in Eq. (4).
2. Any refractive index oscillation in the measurement volume results in a varying angle difference between object and reference beam on the camera sensor and, thus, an oscillation of the modulation depth occurs. This results in an amplitude modulation of the intensity signal in the lower frequency range up to several kHz. When evaluating the instantaneous frequency by performing the Hilbert transform, the low pass part of the signal and the high pass part of interest around the carrier frequency must not overlap in the frequency domain to fulfill Bedrosian’s theorem.

Therefore, a carrier frequency $f_{\text{B}} = 50$ kHz was chosen, allowing the detection of frequency shifts and higher harmonics in the range $\pm \;25$ kHz with a sufficient separation of the bandlimited signal and the low-pass amplitude modulation. The chosen camera frame rate of 200 kHz enables a stable sampling of the described frequency range of interest up to 75 kHz, fulfilling the Nyquist-Shannon sampling theorem. Given the number of 110 × 110 px and the magnification factor of the lens setup L_5_–L_8_, a field of view of 51 × 51 mm^2^ with a spatial resolution of 0.47 × 0.47 mm^2^ per pixel is achieved. The field of view is determined by the size of the optics used to expand the laser beam in the measurement volume. If a larger field of view for larger flames is of interest, lenses with increased diameter have to be installed. This leads to a lower resolution of the measured field, when keeping the same frame rate. A benchmark of the CLIV system using a loud speaker and a swirl-stabilized flame is provided by Gürtler (2016) and Gürtler et al. (2017).

**Fig. 2:**
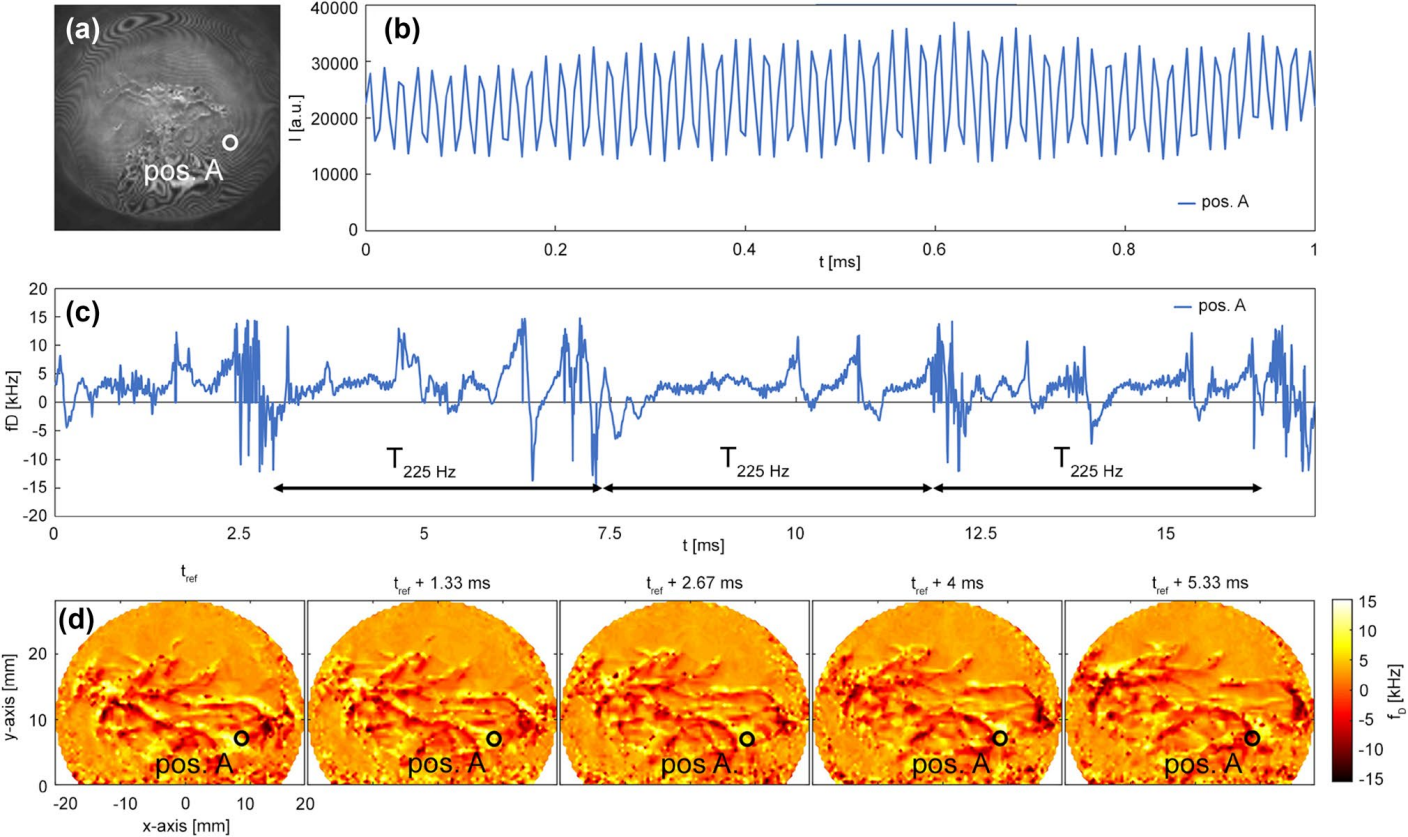
**a** Instantaneous interference pattern recorded with the high-speed camera at a siren frequency of 225 Hz. **b** Shows the time-series of the intensity signal of one pixel at position A, indicated with a white circle in a). The corresponding time-series of Doppler shifts after demodulation of **b** is plotted in **c**. A periodic occurring pattern with a frequency of 225 Hz can be observed. **d** Shows full-field instantaneous Doppler shifts at five consecutive timesteps

As an example, the signal evaluation process of one flame measurement is demonstrated in Fig. 2. In Fig. 2(a) the interference pattern as detected with the camera sensor is depicted. Additionally, Fig. 2(b) shows the signal for one pixel, marked with position A, where the modulation of the intensity can be observed clearly. After performing the demodulation of the intensity signal as described above, the instantaneous frequency of the signal is plotted in Fig. 2(c). For the data shown, the flame was excited at 225 Hz and, thus, an accordingly periodic reoccurring pattern is visible. A full-field time-series of line-of-sight (integral) Doppler shifts can be seen in Fig. 2(d).

## Experimental setup

### Combustor

All measurements were done on a lean, swirl-stabilized methane combustor with a cylindrical confinement under atmospherically conditions. For optical access, a quartz-glass cylinder with a height of 210 mm, an outer diameter of 120 mm and a wall thickness of 3 mm was used. On top of the quartz-glass an additional aluminum cylinder was placed to reach a total combustor height of 616 mm. The aluminum cylinder was choked downstream and adjustable in height.

**Fig. 3:**
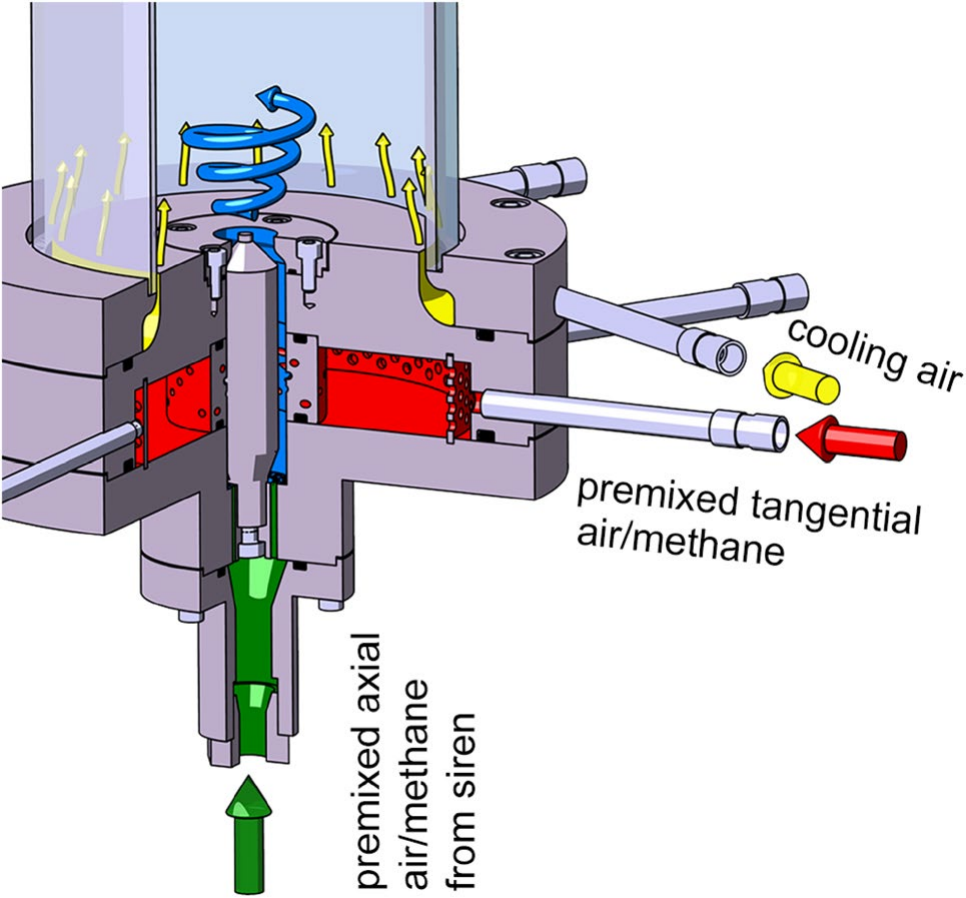
Cut through combustor: both axial and tangential air were already premixed with methane before entering the combustor. Axial air was modulated with a siren. The confinement consisted of a quartz-glass cylinder and an aluminum exhaust with a total height of 616 mm

The combustor consists of three feed lines: premixed axial and tangential supply, in which the axial air/fuel got modulated by a siren (Giuliani et al. 2012) at defined frequencies, and a cooling air supply. The tangential air gets swirled through four rows of eight cylindrical bores, aligned tangentially and symmetric around the burner axis, as shown in Fig. 3. According to Candel et al. (2014) a simplified swirl number of 0.53 was calculated. Assuming complete combustion, the thermal power of the combustor was 3.44 kW, the mean equivalence ratio was 0.88. Fuel and air mass-flows were measured via caloric mass flow meters (EL-Flow, Bronkhorst) and set to the following values: $\dot{m}_{\text{CH4}}$ = 0.068 g/s, $\dot{m}_{\text{ax}}$ = 0.705 g/s, $\dot{m}_{ \tan }$ = 0.628 g/s, $\dot{m}_{\text{cooling}}$ = 1.2 g/s.

### Other optical measurement techniques

#### Background-oriented schlieren method

When observing a distant speckled object through a field of density gradients, the refraction of light causes an apparent displacement of the speckles, proportional to the line-of-sight gradient of the refractive index. With the linear Gladstone–Dale relation, the refractive index can be linked to density. If a gradient-free reference is available, correlation algorithms can be used to calculate the displacement of the speckles. Absolute values can be calculated by integrating the gradients and finally, with a calibration factor the arbitrary displacements can be linked to absolute density. A detailed discussion on the theory and the application of BOS can be found in Hugues and Raffel (2001) and Raffel (2015).

**Fig. 4:**
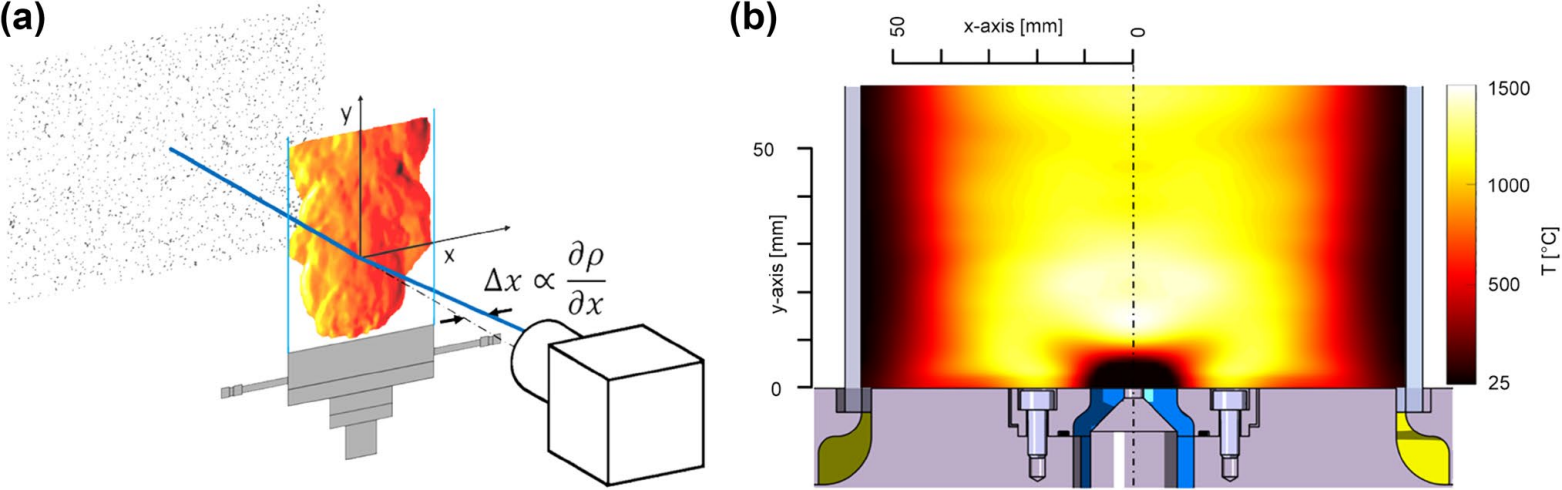
**a** Principle of the BOS setup used. The CCD camera was focused on a speckled background. Density gradients are refracting light and causing an apparent shift of the speckles on the recorded frames. When correlating such frames with a gradient-free reference density gradients and furthermore absolute density can be calculated. **b** Temperature field obtained from BOS measurements at a siren frequency of 225 Hz

The experimental setup for the BOS measurements used is shown in Fig. 4(a). A random dot pattern was used as background, which was placed behind the combustor. Two headlights ensured a uniform illumination, while the aperture of the CCD camera (DMK 31BF03) was set to minimum, so that only beams with a slight angular deviation of the camera axis were recorded. 1000 reference- and flame images were taken with a frame rate of 60 Hz, exposure time 1 ms and a resolution of 1024 × 768 px. The reference images were recorded after the measurements (with hot window glasses), so that the thermal expansion of the cylindrical glass did not influence the results.

#### Spectroscopy

**Fig. 5:**
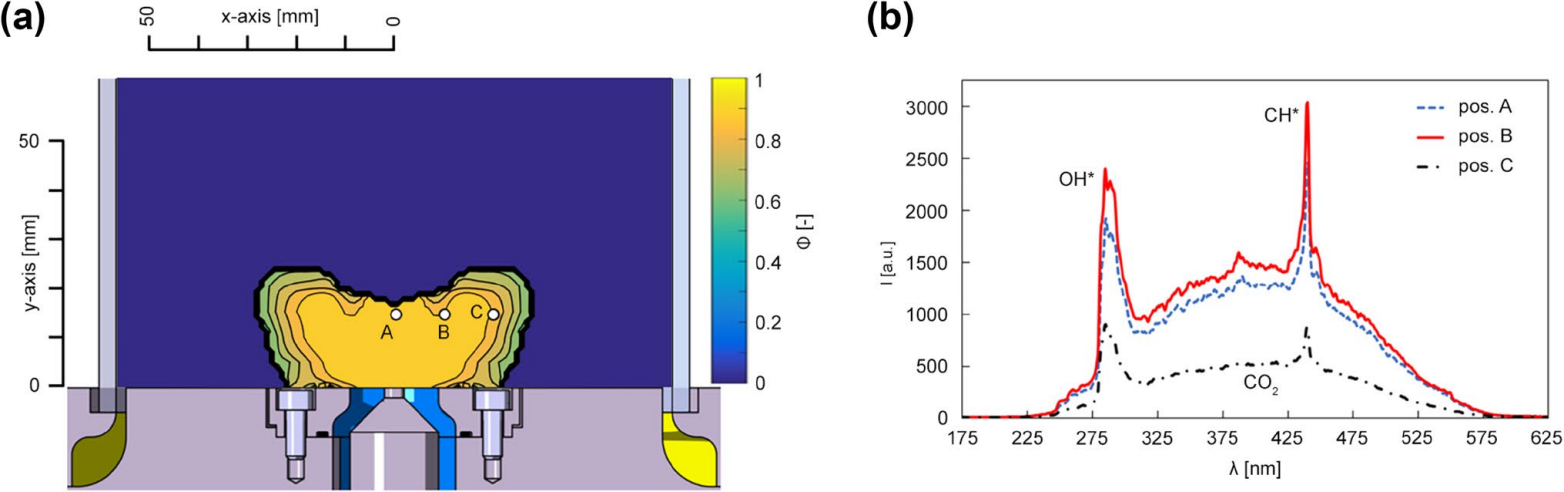
**a** Local distribution of equivalence ratio. In the main reaction zone, the equivalence ratio of the premixed flame was stable with a value of 0.88. At higher radial distances, cooling air entrained the reaction zone. **b** Plots flame spectra at an axial position of 15 mm (y-axis) and three radial positions (A *r* = 0 mm, B *r* = 10 mm, C *r* = 20 mm). OH*-and CH*-peaks are clearly visible, for the calculation of the equivalence ratio a CO_2_ background correction was carried out

Before entering the combustor, air and fuel were already premixed, to ensure a homogeneous mixture. Ideally the equivalence ratio should not change in the combustion zone but since cooling air was necessary, the air/methane mixture got diluted at radial positions close to the glass cylinder. A spectrograph (SpectraPro 2300i, 600 lines per mm grating, 30 mm entrance slit, resolution 3.9 cm^− 1^, Acton) and an ICCD camera (NanoStar) together with an UV lens (105 mm, f/4.5, Nikon) were used to record light emission of the flame from 5 to 35 mm in axial height. 6400 recorded images were averaged for further data processing. Figure 5b shows averaged spectra at three radial positions (A *r* = 0 mm, B *r* = 10 mm, C *r* = 20 mm). The peaks of the OH* radical at 310 nm and CH* at 430 nm are clearly visible, while the absence of Swan-bands from 470 to 550 nm (C*) clearly states a lean combustion, without soot production. To calculate the local equivalence ratio, the CO_2_ background corrected ratio of OH*/CH* radical emission, together with correlation tables of Lauer (2011). Time-averaged results are shown in Fig. 5(a). In the flame center an equivalence ratio of 0.88 was calculated, identical results were obtained from mass flow calculations. Closer to the walls of the quartz-glass cylinder, cooling air diluted the mixture, resulting in an equivalence ratio from approximately 0.7–0.75. Small values of OH* and CH* intensity, superimposed with noise outside the main reaction zone led to random results, therefore masking was applied to the data. Equivalence ratio data were then used to calculate local ratios of heat capacity and Gladstone–Dale constants. Compared to the assumption of a fixed Gladstone–Dale constant, the accuracy can be enhanced in the range of 1–3 percent if local data are available (Eq. 5).

#### Photomultiplier

As reference, a photomultiplier (PMM01, Thorlabs) with an OH* bandpass filter (310 ± 3 nm, Edmund Optics) was placed at a radial distance of 1.2 m from the combustor to record the global emission of OH* radicals. The photomultiplier (PM) recordings were triggered by the CLIV measurements to ensure a synchronous recording of data. For data evaluation, a fast Fourier transform (FFT) of the photomultiplier time signal as well as a cross correlation between photomultiplier and the siren trigger was calculated, to get the global emission spectra and their corresponding phase angles in relation to the trigger signal.

Assuming complete combustion, the mean OH* emission can be used to calculate a calibration factor in Watt per radiant emittance to further calculate the heat release fluctuation spectra in Watts.

## Data evaluation

### CLIV

**Fig. 6:**
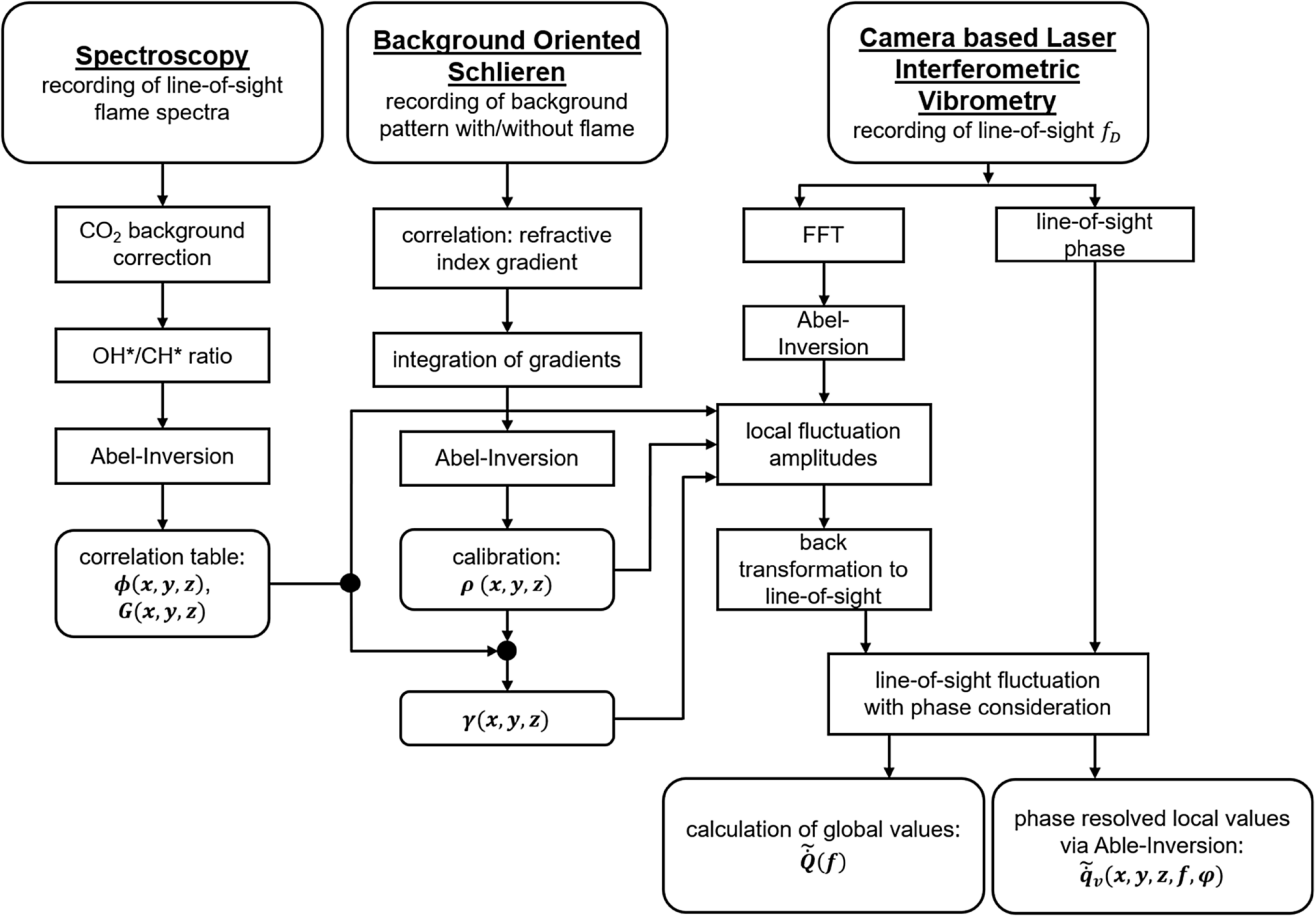
Main signal processing steps for spectroscopy, BOS and CLIV. Spectroscopy and BOS are necessary to provide equivalence ratio $\varPhi$ and mean density $\rho$ for the evaluation of the CLIV data

The evaluation of CLIV data from refractive index fluctuations to local and global heat release fluctuations consists of several steps which will be explained briefly in this chapter. A graphical summary of the main steps is given in Fig. 6. Starting from the time signal of intensity recorded with the high-speed camera, at first, quadrature demodulation technique was applied to the camera intensity signal to calculate the time-dependent Doppler shifts for each camera pixel, as described in Sect. 2.2. In a next step fast Fourier transform (FFT) of the time-dependent Doppler shifts, as well as a cross correlation between Doppler signals and the synchronously recorded siren trigger was calculated for each pixel of the high-speed camera. Such a correlation results in the phase lag between the LIV Doppler signal and the siren for each pixel (‘phase plot’). According to Eq. (5) this Doppler signal is related to the density fluctuations. Therefore, the pixelwise phase information is needed to calculate the global oscillation amplitude from oscillations recorded in a single pixel with amplitude $\tilde{\dot{\rho }}_{\text{LOS}} \left( {x,y,f} \right)$ recorded as integral data (line-of-sight, LOS). The tilde indicates the oscillation amplitude at frequency $f$, excited by the siren.

Before summing up LOS data from all pixels, density has to be calculated from the Doppler shifts (Eq. 5) in a first step, and furthermore heat release oscillations from density oscillations as described by Eq. (2). For both equations the constants—namely the Gladstone–Dale constant $G$ in Eq. (5), and $\bar{\gamma }\left( {x,y,z} \right)$, $\bar{p}\left( {x,y,z} \right)$ and $\bar{\rho }\left( {x,y,z} \right)$ in Eq. (2)—are local values, whereas the Doppler signal is still an integral value along line-of-sight. Thus, local data have to be calculated using tomographic algorithms. In general, multiple projections of the LOS data are necessary to obtain a tomographic reconstruction. In case of an axisymmetric flame, Abel-transformation can be applied to a single projection (Pretzler et al. 1992). This axial symmetry was tested for the confined swirl-stabilized flame presented in Sect. 3.1. The LOS FFT spectra of heat release fluctuations fulfilled this requirement within a few percent for all operation points investigated in this work. For Abel transform a Fourier-series-like expansion from software package IDEA (http://optics.tugraz.at) was used as discussed in Greiffenhagen et al. (2019) and Hipp et al. (2004).

Then, Eq. (5) was applied to the local Doppler shifts to calculate local density fluctuations spectra $\tilde{\dot{\rho }}\left( {x,y,z,f} \right)$. The Gladstone–Dale constant $G$ was set to a fixed value in the combustion zone, since only slight changes of the equivalence ratio occurred in the combustion area, as shown in Fig. 5. Outside the reaction zone $G$ was set to the corresponding value of air with a transition zone between flame and ambient air.

Next, local heat release fluctuation spectra $\tilde{\dot{q}}\left( {x,y,z,f} \right)$ were calculated from density fluctuations $\tilde{\dot{\rho }}\left( {x,y,z,f} \right)$ according to Eq. (2) under the assumptions discussed in Sect. 2.1. Local mean density $\bar{\rho }\left( {x,y,z,f} \right)$ was calculated from BOS measurements as discussed in the next section in detail. Different siren excitation frequencies resulted in slight changes of flame shape and temperature distribution, hence BOS recordings were done for all operation points investigated with CLIV. For precise calculation of heat release oscillations especially the mean density has to be measured as accurate as possible. This is due to the fact that the inverse of the mean density is a weighting factor to density fluctuations as given by Eq. (2). It was not possible to synchronously record refractive index fluctuations with CLIV and gradients thereof with BOS due to the dimensions of the CLIV measurement setup. Thus, it had to be assumed, that mean density (and the respective temperature distribution) did not change between the two separate CLIV and BOS measurements, as long as the same operation parameters are used. Pressure was constant and ambient. Local $\bar{\gamma }\left( {x,y,z} \right)$ was calculated from temperature (BOS) and equivalence ratio (spectroscopy).

It is not possible to directly add LOS phase information—obtained by correlation of the LOS Doppler signal to the siren signal—to local heat release data, because no tomographic reconstruction of LOS phase lags is possible. Therefore, LOS phase spectra $\varphi_{\text{LOS}} \left( {x,y,f} \right)$ have to be linked to the LOS amplitude spectra $\tilde{\dot{q}}_{\text{v,LOS}} \left( {x,y,f} \right)$ to describe the oscillations by a complex number $\tilde{\dot{q}}_{\text{v,LOS}} \left( {x,y,f} \right) e^{{i\varphi_{\text{LOS}} \left( {x,y,f} \right)}}$. Therefore, local (radial) data $\tilde{\dot{q}}\left( {x,y,z,f} \right)$ were used to form a data volume by rotation around the vertical axis, with a line-of-sight integration afterwards to form the projection data $\tilde{\dot{q}}_{\text{v,LOS}} \left( {x,y,f} \right)$. From this complex number for each pixel the global heat release fluctuations were calculated to obtain the global heat release

6 $$\underset{\raise0.3em\hbox{$\smash{\scriptscriptstyle-}$}}{\tilde{\dot{Q}}} \left( f \right) = \mathop \sum \limits_{x} \mathop \sum \limits_{y} \tilde{\dot{q}}_{\text{v,LOS}} \left( {x,y,f} \right) e^{{i\varphi_{\text{LOS}} \left( {x,y,f} \right)}}$$

with $x$ and $y$ the pixelwise coordinates of the camera frame.

It is important to note, that contrary to amplitude spectra $$\tilde{\dot{q}}_{\text{v,LOS}} \left( {x,y,f} \right)$$, phase $$\varphi_{\text{LOS}} \left( {x,y,f} \right)$$ did not always show axisymmetric behavior, when the flame was excited at frequencies aside of the natural resonance frequency of the combustor (about 225 Hz). To consider the interference between left and right side of the flame when calculating global values, the full data field containing phase information must be used to solve Eq. (6), and not only the half plane which is output of an Abel transform. The magnitude of the global heat release oscillations at a given frequency is provided by $$\left| {\underset{\raise0.3em\hbox{$\smash{\scriptscriptstyle-}$}}{\tilde{\dot{Q}}} \left( f \right)} \right|$$, while the angle $$\varphi \left( f \right)$$ presents the phase delay of the global oscillation in relation to the siren trigger with respect to the 2π ambiguity.

Local data are now also accessible from the complex LOS oscillations on the right side of Eq. (6). To obtain local information for the single phase steps, the real part of the LOS signal $\tilde{\dot{q}}_{\text{v,LOS}} \left( {x,y,f} \right) e^{{i\varphi_{\text{LOS}} \left( {x,y,f} \right)}}$ is Abel transformed again. With a constant phase delay $\Delta \varphi$ added to the measurement positions $\tilde{\dot{q}}_{\text{v,LOS}} \left( {x,y,f} \right) e^{{i(\varphi_{\text{LOS}} \left( {x,y,f} \right) + \Delta \varphi )}}$ before calculating the Abel inversion of the real part of the LOS signal, the resulting data shows the local distribution of heat release fluctuations at single time or phase steps relative to the siren trigger. In the present work, one full period was split into 16 phase steps.

### Background-oriented schlieren method

After recording single BOS images, a correlation between image frames (with flame) and reference frames (without flame) was carried out for each of the 1000 recordings, using commercially available particle image velocimetry software (Dantec DynamicStudio, Version 6.2). The interrogation area was set to 16 × 16 px with a vertical and horizontal overlap of 50 percent, thus a resolution of 1.75 × 1.75 mm^2^ for the results was achieved. Next, the gradients were integrated line-wise for each frame in horizontal direction (x-direction) to provide density information, which was then averaged over the 1000 frames recorded. To compensate offset errors, integration was not only performed from left to right but also the opposite direction with both results averaged.

The resulting data describe density proportional LOS values. Again, local data have to be calculated from LOS data by Abel transform, since the time-averaged density gradient data also showed axisymmetric behavior. For all Abel transforms performed in this work, the local data were integrated along the LOS direction (z-direction) and compared to the original LOS data recorded to verify the parameters used for Abel transform.

The resulting local BOS data provide qualitative local density data. Thermocouple measurements in the confinement while operating the test rig provided information on local temperatures. Information which was then used to calibrate density via the ideal gas equation. Due to the size of the BOS interrogation area, BOS data resulted in a coarser grid than that resulting from the CLIV recordings. Hence it was necessary to interpolate data to the corresponding CLIV measurement grid.

## Results and discussion

The results presented in the following sections are intended to follow the chronological structure of data evaluation visualized in Fig. 6, beginning with instantaneous recordings of density fluctuations, to local phase-averaged data and finally to global oscillation amplitudes. Therefore, in a first step unsteady data of an ignition process are presented to demonstrate the potential of CLIV to capture dynamical processes by spatio-temporal correlations. In a second step, thermoacoustic oscillations in a lean, confined, premixed and swirl-stabilized methane flame were forced by a siren to obtain a better signal-to-noise ratio when detecting oscillation amplitudes at single frequencies. Data evaluation as described in Sect. 4.1 was applied to data obtained from CLIV recordings of the forced flame, a corresponding time-series of raw data with siren excitation at 225 Hz is depicted in Fig. 2(d).

### Instantaneous recordings of density fluctuations

**Fig. 7:**
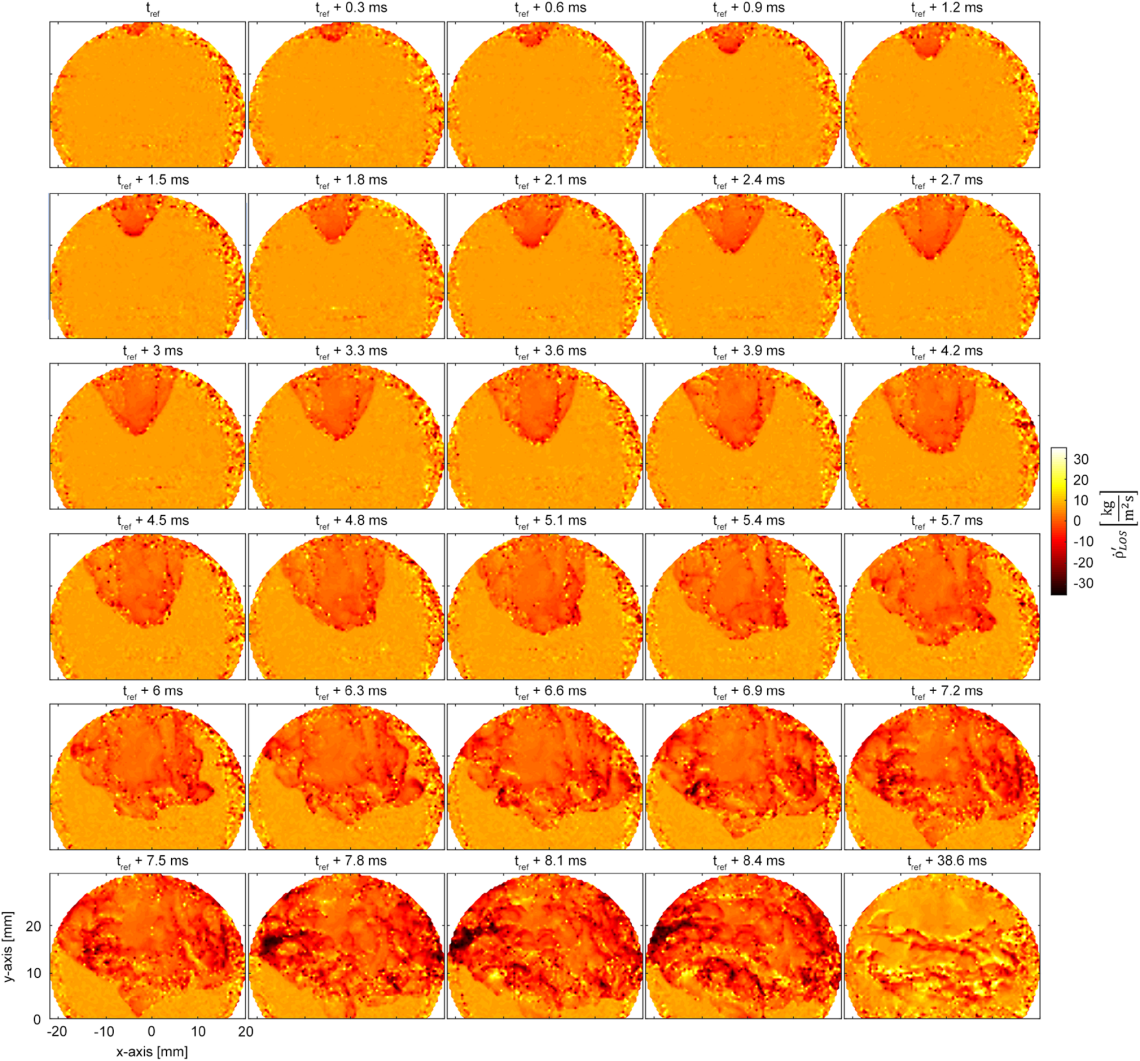
Line-of-sight unsteady data of an ignition process with a temporal resolution of 300 µs. The last image shows a stabilized flame 38.6 ms after the flame front enters the measurement volume

If no spectral analysis is performed on the time-series of Doppler shifts, CLIV can visualize unsteady processes with a temporal resolution of 5 µs. In the following a highly unsteady ignition process is shown, hence the assumption of an axisymmetric behavior does not hold true anymore and in contrast to time-averaged data, no Abel-transformation can be carried out. Therefore, with LOS density fluctuations a fixed Gladstone-Dale constant for the whole field of view has to be used in Eq. (2). Figure 7 shows the resulting data, the time-step between the frames is 300 µs, thus every 60th frame is plotted. The elliptically shaped field-of-view is caused by masking, which had to be applied due to a low modulation depth (Eq. 4) in the image corners. The fluctuations plotted are normalized to kg/m^2^s for each camera pixel.

To record the data shown in Fig. 7, the combustor was filled up with an air/fuel mixture for a few seconds, before the gas mixture was ignited at the combustor exit, approximately 600 mm downstream of the burner nozzle. Until *t*_ref_ + 3.9 ms the flame front propagates undisturbed upstream, in and behind the flame front the hot, burnt gases are clearly visible due to rapid changes in density. Starting at *t*_ref_ + 4.2 ms the swirling air/fuel flow at the burner outlet is rolling up the flame front, which then reaches the burner nozzle. At *t*_ref_ + 6.9 ms the flame front attaches to the ring-shaped burner nozzle and a full twirl of the flame front can be observed. It starts on the left side, then moves to the right and back again. Finally, the remaining fuel distributed in the combustion chamber gets consumed. About *t*_ref_ + 40 ms the flame detaches from the burner nozzle and gets stabilized by the swirling flow. Thus, the CLIV technique has the potential to investigate the interaction between propagating flame front and flow velocity similar to density-tagging velocimetry (Raffel et al. 2011), previously only possible by FMDGV or similar tracer-based techniques (Schlüßler et al. 2015).

### Lean, confined, premixed and swirl-stabilized methane flame

#### Phase-averaged local heat release oscillations

**Fig. 8:**
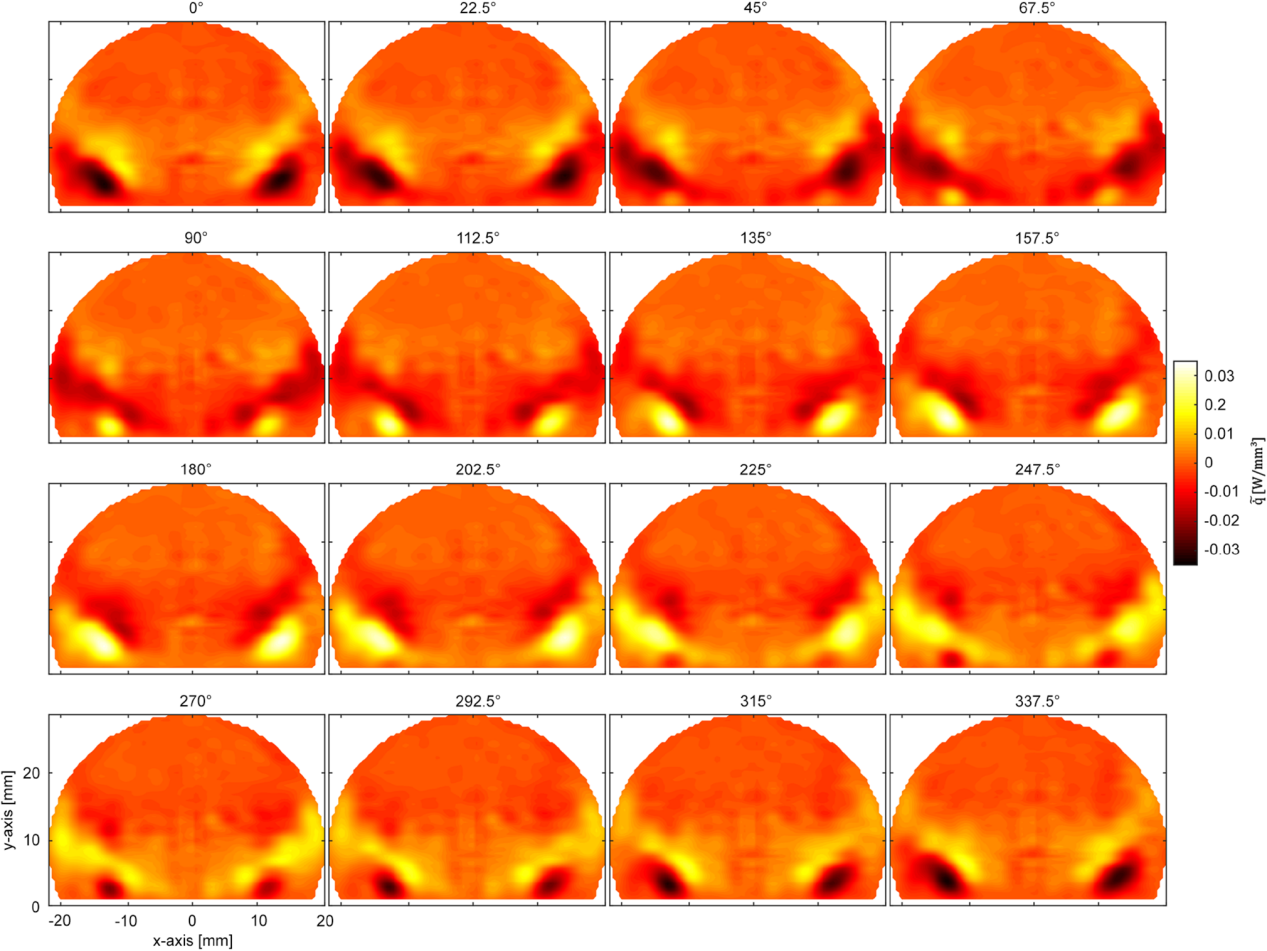
Phase-averaged and local heat fluctuations at 225 Hz, calculated via Abel-transformation from line-of-sight data. One period of fluctuations was split into 16 phase angles, to show the propagation of oscillations downstream through the combustor

To calculate phase-averaged local data of heat release oscillation, all evaluation steps described in Sect. 4.1 and shown in Fig. 6 must be carried out. Phase averaging means that each frame is assigned an oscillation cycle phase, based on the trigger signal from the monofrequent acoustic excitation (siren). For phase-averaged data the averaging process is performed only for the data associated with one cycle phase. Instead of assigning and sorting images, this process can be performed via fast Fourier transform by combination of LOS phase spectra $\varphi_{\text{LOS}} \left( {x,y,f} \right)$ and LOS amplitude spectra $\tilde{\dot{q}}_{\text{v,LOS}} \left( {x,y,f} \right)$ as discussed in Sect. 4.1. Figure 8 shows 16 time steps of phase averaged heat release fluctuations for one period at a siren frequency of 225 Hz, by plotting the real part of the Abel transformed LOS heat release oscillations: ${\text{ABEL}}\left\{ {{\text{real}}\left( {\tilde{\dot{q}}_{\text{v}} \left( {x,y,f} \right) e^{{i \left( {\varphi \left( {x,y,f} \right) + \Delta \varphi } \right)}} } \right)} \right\}$. The oscillations are normalized to W/mm^3^ at 225 Hz.

#### Global density oscillations and their relation to oscillations in heat release

To verify the results discussed, a photomultiplier recorded OH* chemiluminescence simultaneously to CLIV as a reference. Heat release fluctuations in turbulent flames are highly complex phenomena, so one must keep in mind, that OH* chemiluminescence has to be interpreted with care, but it is generally accepted that this number does represent global oscillations in perfectly premixed methane flames quite well (Lauer 2011).

CLIV and photomultiplier recordings were carried out for siren frequencies from 125 to 400 Hz in 25 Hz steps. At lower siren frequencies, the flames’ response to the excitation was low, thus at these frequencies both measurement methodologies suffered from a bad signal-to-noise ratio.

**Fig. 9:**
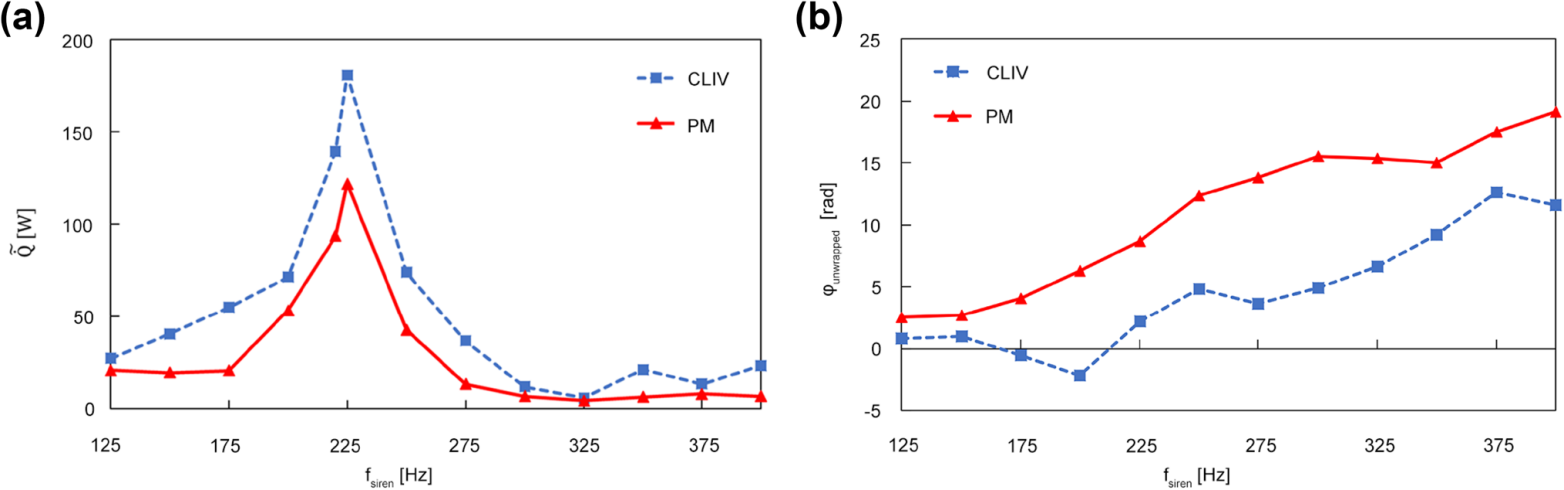
**a** Comparison of the transfer function of global heat release fluctuations measured with CLIV and OH*-chemiluminescence. **b** Comparison of unwrapped global phase angles

**Fig. 10:**
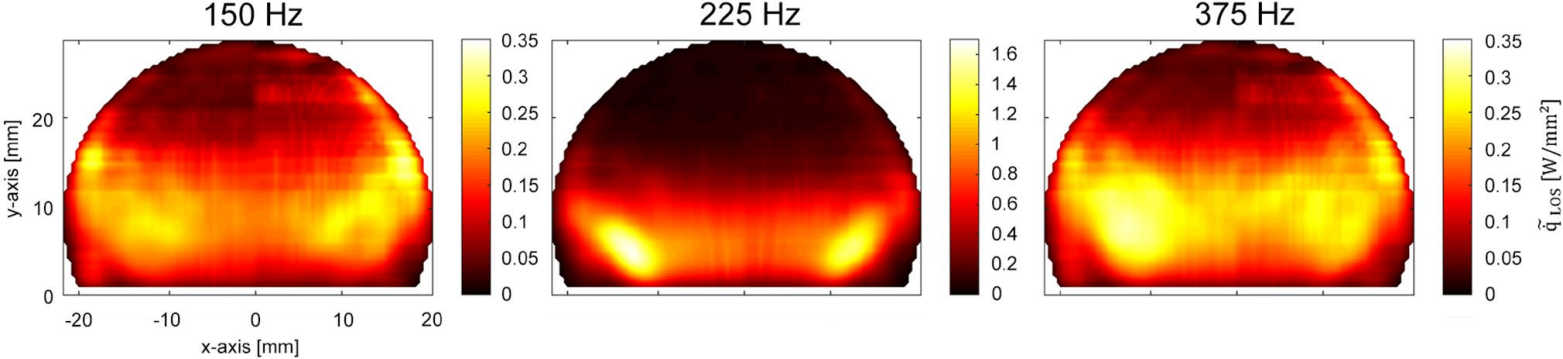
Line-of-sight amplitudes of heat release fluctuations with siren excitation at 150 Hz, 225 Hz and 375 Hz

A comparison of the global heat release oscillation amplitudes $\tilde{\dot{Q}}\left( f \right)$ recorded with the photomultiplier as well as with CLIV can be seen in Fig. 9(a). Qualitatively the two experimental methodologies, based on completely different mechanisms, correlate well. Both show maximum heat release oscillations at the combustor resonance frequency around 225 Hz. When taking a quantitative look at the data, CLIV measurements show a higher amplitude than the photomultiplier over nearly all frequencies. Two main reasons were found to explain this behavior. One was addressed to the field-of-view, which was limited by the optics used and by the signal-to-noise ratio, whereby areas with low SNR had to be masked. Measurements at all frequencies, especially at siren frequencies lower than 200 Hz and higher than 250 Hz showed a radial extension wider than the recorded field size. This not only leads to erroneous results because not all fluctuations were detected, but also Abel inversion requires data fields which are at least close to zero at the maximum recorded radial positions, otherwise the reconstruction of local values results in too high values. Close to the combustor resonance frequency around 225 Hz the heat release fluctuations are more compact as can be seen in Fig. 10 and therefore systematic errors in the calculation of local data are smaller.

However, the general offset of heat release fluctuations obtained from CLIV compared to that of the photomultiplier cannot be explained only by a too small field-of-view of the system. Especially at the resonance frequency the discrepancy is much too large.

To ensure a correct measurement of density fluctuations by CLIV, also recordings with a commercially available digital, single-point LIV (interferometer head OF-503, velocity decoder OFV-5000, calibration factor 1 mm/s/V, 100 kHz bandwidth, no filters, Polytec, Waldbronn, Germany) were performed and used as reference. With this single-point LIV, half of the flame was scanned with a measurement grid of 2 mm and a field size of 30 mm in radius and 60 mm in height. The siren frequency was set to 220 Hz. A detailed instruction on the data evaluation used for single-point LIV used to detect thermoacoustic oscillations quantitatively is given in Greiffenhagen et al. (2019). In the present work, LIV data were then compared to the CLIV data recorded at the same point of operation for the confined flame. Both CLIV and LIV measurements were evaluated using the parameters discussed above and resulted in nearly the same oscillation amplitudes (135 W LIV, 137 W CLIV), while 98 W were calculated from OH* chemiluminescence recordings, using the photomultiplier. While LIV and CLIV will reveal local density fluctuations although these are not primarily caused by fluctuations in heat release, chemiluminescence imaging will show nothing in the absence of chemical reaction.

**Fig. 11:**
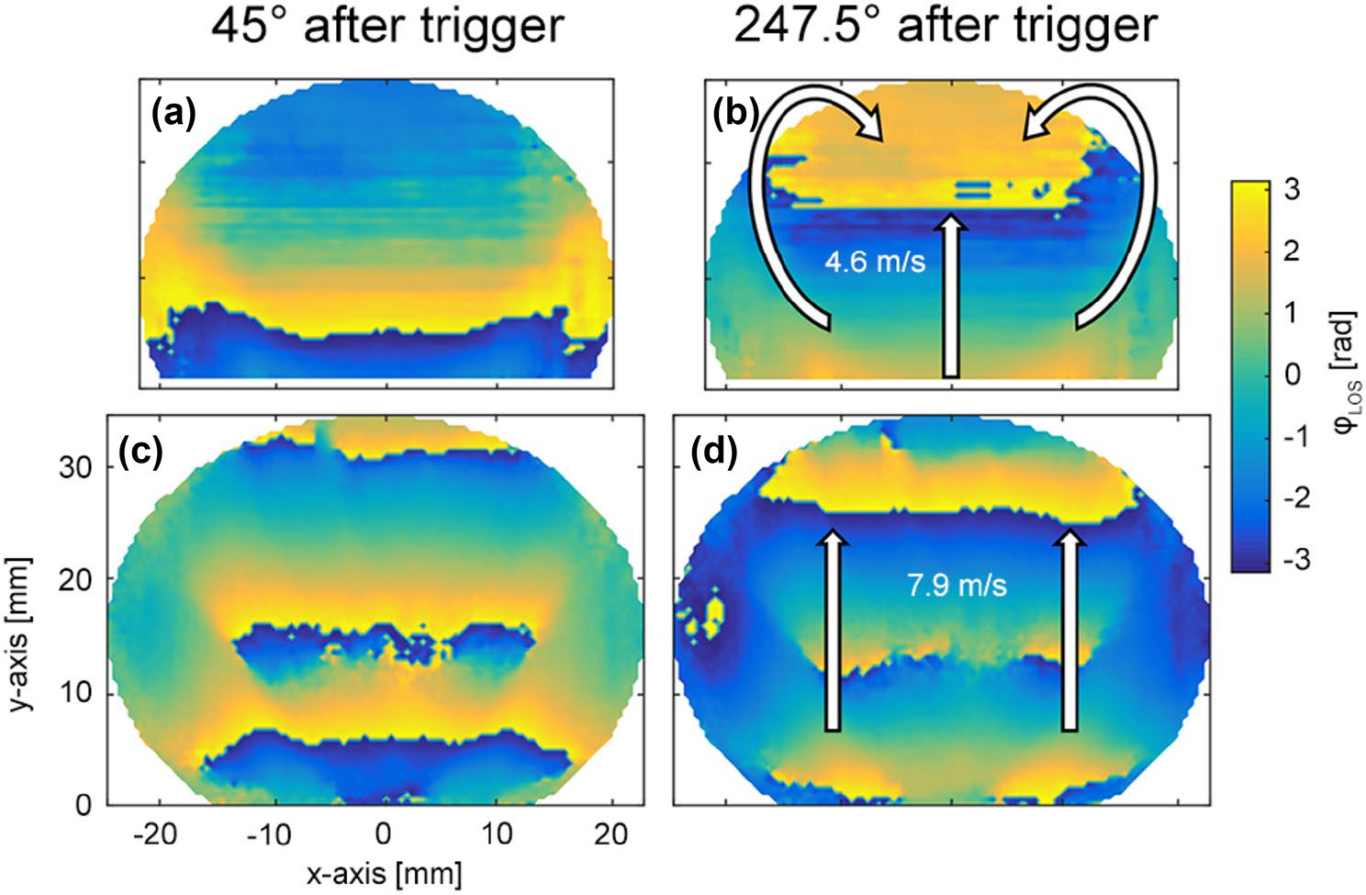
Line-of-sight phase angles referenced by the siren trigger at 220 Hz for the confined flame in **a** and **b**, and for the unconfined flame in **c** and **d**. The deformation of the phase-front in the confined flame (**a** and **b**), indicates a strong recirculation zone. The unconfined flame shows nearly no deformation while the oscillations propagate downstream

An explanation for the discrepancies was found, when the phase plots of the confined flame were compared to that of the unconfined flame. Those plots can be seen in Fig. 11, whereas a and b show the confined flame, while the unconfined flame is plotted in (c and d). When looking at the data of the confined flame, at 45° after the trigger (Fig. 11a), a M-shaped distribution of phase is visible, visualized by a modulo 2π phase step. Later, at 247.5° after the trigger (Fig. 11b) recirculation deforms these phase-fronts (wave-fronts of constant phase). While in the unconfined case shown in Fig. 11c and d, no deformation of the phase distribution can be observed. This indicates a much stronger recirculation of hot gases in the confined case. Also, when taking the propagation of constant phases wave-fronts over time as an estimation of convection velocity, the confined flame shows a lower bulk velocity (~ 4.6 m/s) than the unconfined flame (~ 7.9 m/s), indicating a stronger recirculation in the confined case and a longer residual time of hot gases in the measurement volume. Beside local oscillations in heat release, it also records density gradients which travel with the flow and dissipate downstream. Hence, convective transport of hot and cold spots and recirculation of combustion products in the combustion zone can contribute significantly to the density fluctuations measured with CLIV. Also cooling air which may get entrained into the combustion products can lead to density gradients. In such cases, the convective term can no longer be neglected. Chemiluminescence is not affected by this mechanism, because OH* emission only occurs in the presence of chemical reaction, representing fluctuations in heat release only. If global OH* chemiluminescence and CLIV density fluctuations are recorded simultaneously, the contribution of other effects than oscillations in heat release causing oscillations in density can be quantified, all data important for thermoacoustics as discussed by Eq. (3).

Besides global heat release fluctuations, also the unwrapped phases for OH* chemiluminescence and CLIV are plotted from 125 to 400 Hz siren excitation. But it has to be clear that the phases can only be equal if density fluctuations caused by heat release fluctuations dominate over density fluctuations from convection of non-reacting zones with temperature gradients, thus the results show large discrepancies.

## Conclusion and outlook

A camera-based full-field LIV system (CLIV) was applied successfully to a lean, confined, premixed and swirl-stabilized methane/air flame. The system detects fluctuations of refractive index along line-of-sight of the laser beam with a sample rate of 200 kHz and a resolution of 0.47 × 0.47 mm^2^. The assumptions and algorithms necessary to calculate heat release oscillations from oscillations in the refractive index were discussed in detail. It was found that effects like radiation and pressure fluctuations on density fluctuations are at least two orders of magnitude smaller than that of heat release fluctuations in the combustion zone.

Discrepancies in time-averaged global heat release fluctuations, calculated from CLIV data and OH* chemiluminescence were mainly caused by convection and recirculation of hot and cold spots into the measurement volume. In this case CLIV not only records changes in density caused by heat release fluctuations but also convection of zones with temperature gradients into the measurement volume. In addition, a larger field-of-view for CLIV, especially in lateral direction, would lead to more precise results. Therefore, a large enough lens system is needed.

Global phase angles of CLIV and OH* chemiluminescence data showed large discrepancies. When comparing this data, it has to be clear that the phases can only be equal if density fluctuations caused by heat release fluctuations dominate over density fluctuations from convection of non-reacting zones with temperature gradients. It was already shown in heat release data that that was not the case in the flame investigated.

The main advantage of the CLIV system lies in the fast recording of full-field density fluctuation data with high spatial and temporal resolution. Scanning the same area with a commercially available LIV system would last for 81 h of pure measurement time, while it takes only 63 s with CLIV. The general advantage of CLIV is that all density fluctuations are captured, independent of their cause. By combining CLIV and chemiluminescence data, it is possible to show the non-reacting part of the density fluctuations.

Recording the flame from different projection angles can be used for tomographic reconstruction of complex shaped, non-axisymmetric density fluctuations. Simultaneous recordings of several CLIV systems aligned at different observation angles would even allow three-dimensional reconstruction of unsteady heat release with high temporal and spatial resolution.
